# A Good Woman’s Thoughts

**Published:** 1875-11

**Authors:** 


### A GOOD WOMAN'S THOUGHTS.

We print below a letter from a lady of our acquaintance, and wish to remark that we shall be glad to hear from her in the same line, each month. She shall have have all the space she wants, and so shall those good ladies who may write us in accordance with her request.

DEAR DOCTOR:

I have read your journal with great interest for many years, and I like it. You give us many excellent maxims, and point out clearly just where the best things can be bought, and it was truly a pleasure to see your kindly face beaming upon us from the cover of the October number. After looking steadily in your eye for some moments I drew encouragement from its expression, and determined to carry out a resolution, long ago half formed, to write you a letter to ask you if you would open your columns to discussions on subjects of interest to women, who find it so hard even in these enlightened days, to find a hearing. I am a housekeeper, and I want to be a good one. I treasure up all the hints I get from the various books, magazines and newspapers that come in my way, and I must express through you the gratitude I feel towards Cousin Kate for the many new suggestions she has given me in her admirable story. Her processes are stated with such perfect accuracy that the result is always sure to be just what she foretells, and thus cooking that has been a weariness to the flesh, becomes a pleasure and delight. But I have often felt that understading this subject so completely, she must be equally competent to treat of other household matters, and I have wished, when some of my perplexing days have come, that I could in some way reach her, for I am sure she knows exactly how to plan everything in the best possible manner and to meet and provide for whatever emergencies arise.

Then, Dr., you have my gratitude especially in another direction. My husband has been agitated ever since I knew him, and I presume many long years before, upon the boot question. Lasts have been made for him on most of the anatomical models of the day, but he has never found full satisfaction till through your columns he was introduced to the McComber system. Not over sanguine at first, I think he was rather persuaded by my solicitations to try once more, and now instead of giving weeks to the "breaking in" of a pair of new boots he wears them the whole of the first day without a particle of inconvenience or a thought that the old ones are gone. For this I thank you; but I have not time to enumerate all the valuable helps I have received the past year; let this suffice for the present.

We have all heard the three maxims of Miss Hamilton—

"Do everything in its proper time."
"Keep everything to its proper use."
"Put everything in its proper place,"

and every housekeeper will see at once how much her duties would be simplified if these rules were strictly followed: but even with this systematic arrangement much remains to be done, for the world is constantly growing wiser, and easier ways of performing the ever recurring labors of the household are continually coming to our notice. The great question seems to be how to teach the multitude of women who are interested in these matters, the best and simplest way to provide an attractive home for those dear to them. I believe if your readers, who have, no doubt, many ways of their own for securing the most perfect results in various directions, would exchange ideas upon these subjects, much mutual good might be wrought. For instance, when I took out my furs the other day I longed to tell every lady in the land in what perfect condition I found them, and how little trouble it had been to ensure their safety. The latter part of April I exposed them to a great degree of heat and then wrapped them carefully in newspapers and laid them away in a trunk where they have been calmly sleeping through the long summer days, and now, as I shake them out, they really seem to look brighter and fresher for their retirement. I have lost furs in previous years, simply I believe from leaving them around too long in the spring, so that the eggs were deposited by the busy little millers; and this having happened, no camphor or powders will prevent the moths from eating our valuables. Heat will destroy these eggs I believe, and moths are not fond of printer's ink: and it may be that the careful wrapping, completely excluding the chance of even a pin-hole, prevented the entrance of the creatures; hence the safety of the furs was secured.

And then, although these hints are so useful in their place, it seems to me it would be very pleasant to know what women are doing in homes remote from our busy city. In return they might like to hear something of our amusements, literary entertainments, etc., and I wish if any one has a word of encouragement or suggestion to give, you would allow her to do so through your magazine.

I read the other day, with a great deal of interest, in that very valuable column of the Weekly *Tribune*, entitled Home Interests, of a society in Boston for the continuance of study after one's school days are over, and I felt that I would like to learn a great deal more concerning it, but I did not know just how to obtain the information, and I often want to get at something which I cannot reach by way of private correspondence, and so I would like your advice on this and kindred subjects.

Suppose then I send you from time to time such domestic items as please me. Of course you will use your own judgment in regard to publishing them, but I fancy I could once in a while offer a suggestion worth reading, and I shall be grateful if you will give me the opportunity. I have now a little string of random thoughts which I will append and you shall print such as you deem valuable and consign the rest to that oblivion which ever awaits the crude compositions of the unskilled writer—the waste basket.

